# Denmark's sharp rise in the annual prevalence of gestational diabetes: Rethinking screening and prevention

**DOI:** 10.1111/aogs.70050

**Published:** 2025-08-27

**Authors:** Fereshteh Baygi, Christina Anne Vinter, Jens Søndergaard

**Affiliations:** ^1^ Research Unit of General Practice, Department of Public Health University of Southern Denmark Odense Denmark; ^2^ Research Unit for Gynecology and Obstetrics, Steno Diabetes Center Odense, Odense University Hospital University of Southern Denmark Odense Denmark

Denmark has historically reported relatively low gestationel diabetes mellitus (GDM) rates (3%–4% of pregnancies).^1^ However, recent data show a concerning rise.^2^ A national cohort study of over 287 000 births between 2013 and 2017 showed an average 7% annual increase in GDM prevalence, reaching 4.2% nationally by 2017, with some regions approaching 6.2%.^2^ This upward trend is alarming and warrants immediate evaluation, as Denmark's previously low GDM rates may soon align with higher rates observed in other countries. This shift carries serious implications, including increased risks of macrosomia, childhood obesity, and the future development of type 2 diabetes.^3^

This increase has occurred despite unchanged screening criteria, indicating a shift in maternal health risk profile. Among the modifiable risk factors contributing to this trend, rising maternal age and pre‐pregnancy body mass index (BMI) are well established.^3^ For instance, women aged 35 to 49 have nearly double the GDM prevalence of those aged 25 to 34.^1^ Furthermore, women of non‐Western origin face a significantly higher risk (about 1.7 times greater) compared to native Danish women.^1^ These demographic shifts contribute to a growing burden on maternal health services, as they are associated with higher GDM risk and often require more individualized screening, care coordination, and follow‐up.

Denmark employs a risk‐factor‐based screening approach, in which only women with predefined criteria receive an oral glucose tolerance test. These criteria include pre‐pregnancy BMI ≥ 27 kg/m^2^, previous GDM, first‐degree relatives with diabetes, polycystic ovary syndrome (PCOS), twins or multiple pregnancies, and previous delivery of a macrosomic infant (≥4500 g), and glucosuria at any stage of pregnancy.^4^ If glucosuria is detected, an OGTT is prompted unless a normal test was performed within the past 4 weeks.^4^ Additionally, notably, maternal age and ethnicity are not part of these predefined criteria. While this model is resource‐conserving, it may fail to identify a significant number of GDM cases, resulting in a substantial gap in detection. This is supported by recent Danish data showing that if WHO 2013 diagnostic thresholds were applied, the estimated GDM prevalence would rise from 2.2% to 21.5%, identifying many previously undiagnosed women at elevated risk of adverse outcomes.^5^ As more women meet at least one existing risk factor—such as elevated pre‐pregnancy BMI—the current approach loses its intended selectivity and may not function effectively as a targeted screening strategy. Moreover, employing diagnostic thresholds that are less stringent than those recommended by WHO 2013 means that many milder cases go undetected.^5^, ^6^ Evidence indicates that even mild hyperglycemia can increase adverse outcomes, and that treatment can improve both maternal and neonatal health.^6^ This calls into question the continued appropriateness of the current screening policy.

Universal screening for GDM is currently recommended for 24 to 28 weeks of gestation.^7^ Revising screening criteria such as lowering BMI or age threshold may enhance case detection by identifying women who would otherwise remain undiagnosed.^5^ While such changes would require additional resources, they also highlight how diagnostic definitions directly shape reported prevalence and clinical action.

The rising prevalence of GDM and existing detection gaps present both clinical and policy challenges. Undiagnosed or late‐diagnosed GDM increases the risk of pregnancy complications, such as large‐for‐gestational‐age infants and shoulder dystocia.^8^ GDM in general has long‐term metabolic consequences: Mothers face an elevated risk of T2D,^3^ while offspring are more likely to develop obesity, metabolic syndrome, and impaired glucose tolerance.^9^ This underscores the need for continued postnatal monitoring and support. Although national guidelines to improve GDM detection are currently pending approval, both their timely implementation and prevention measures must be prioritized.

Pre‐conception care should target modifiable risk factors and engage high‐risk women early. Women with GDM require structured follow‐up after delivery; however, adherence remains low.^10^ Integrating postpartum glucose testing and long‐term monitoring into primary care could improve outcomes and reduce long‐term complications.

Future analyses using Danish data to estimate population‐attributable risk could further guide these efforts by quantifying the broader impact of GDM and improving identification of high‐risk individuals.

We believe that Denmark's rise in GDM prevalence is no longer negligible. Previously distinguished by its low rates, the country is now moving closer to the European average. A more integrated, data‐driven approach covering preconception to postpartum care is essential—using registry data and risk profile to guide prevention, screening, and follow‐up. Timely prevention and screening can mitigate the long‐term consequences for women and future generations.

## AUTHOR CONTRIBUTIONS

FB conceptualized the editorial, conducted the literature review, and wrote the firts draft. JS, and Vinter C.A provided critical review. All authors approved the final version for publication.

## CONFLICT OF INTEREST STATEMENT

None declared.

## Data Availability

Data sharing not applicable to this article as no datasets were generated or analysed during the current study.
